# Nanoparticles exhibiting self-regulating temperature as innovative agents for Magnetic Fluid Hyperthermia

**DOI:** 10.7150/ntno.55695

**Published:** 2021-03-15

**Authors:** Marco Gerosa, Marco Dal Grande, Alice Busato, Federica Vurro, Barbara Cisterna, Enrico Forlin, Filippo Gherlinzoni, Giovanni Morana, Michele Gottardi, Paolo Matteazzi, Adolfo Speghini, Pasquina Marzola

**Affiliations:** 1 Department of Diagnostics and Public Health, University of Verona, Piazzale L.A. Scuro, 37134 Verona, Italy.; 2 Nanomaterials Research Group, Department of Biotechnology, University of Verona and INSTM, RU Verona, Strada Le Grazie 15, I-37134 Verona, Italy.; 3 Department of Computer Science, University of Verona, Strada Le Grazie 15, 37134 Verona, Italy.; 4 Department of Neurosciences, Biomedicine and Movement Sciences, University of Verona, Piazzale L.A. Scuro 10, 37134 Verona, Italy.; 5 MBN Nanomaterialia S.p.A., Via Giacomo Bortolan, 42, 31050 Carbonera Treviso, Italy.; 6 Foundation for Nanotheranostics Research in Cancer Therapy, RNC, Treviso, Italy.

**Keywords:** Magnetic Fluid Hyperthermia, Self-regulating temperature, Curie temperature, Nanoparticles, MRI.

## Abstract

During the last few years, for therapeutic purposes in oncology, considerable attention has been focused on a method called magnetic fluid hyperthermia (MFH) based on local heating of tumor cells. In this paper, an innovative, promising nanomaterial, M48 composed of iron oxide-based phases has been tested. M48 shows self-regulating temperature due to the observable second order magnetic phase transition from ferromagnetic to paramagnetic state. A specific hydrophilic coating based on both citrate ions and glucose molecules allows high biocompatibility of the nanomaterial in biological matrices and its use *in vivo*. MFH mediator efficiency is demonstrated *in vitro* and *in vivo* in breast cancer cells and tumors, confirming excellent features for biomedical application. The temperature increase, up to the Curie temperature, gives rise to a phase transition from ferromagnetic to paramagnetic state, promoting a shortage of the r_2_ transversal relaxivity that allows a switch in the contrast in Magnetic Resonance Imaging (MRI). Combining this feature with a competitive high transversal (spin-spin) relaxivity, M48 paves the way for a new class of temperature sensitive T_2_ relaxing contrast agents. Overall, the results obtained in this study prepare for a more affordable and tunable heating mechanism preventing the damages of the surrounding healthy tissues and, at the same time, allowing monitoring of the temperature reached.

## Introduction

Cancer is a serious health issue due to a large number of cancer-related human deaths worldwide. Nowadays, conventional approaches commonly used for cancer treatment include a combination of chemotherapy drugs, invasive surgery, guided focused ultrasound ablation, thermal ablation, and radiation therapy. However, none of these techniques is free from risks due to the possible high level of toxicity and invasiveness.

Nanotechnology has provided valuable tools in the war against cancer during the last few years, including systems for selective drug and gene delivery or innovative diagnostic agents 1, 2, 3. Nanotechnology advancements have also made possible developing a therapeutic approach named Magnetic Fluid Hyperthermia (MFH) based on local heating of tumor cells. MFH is considered a promising cancer treatment method, known as green therapy due to the limited effect on the tumor surroundings and relatively low toxicity 4. MFH is currently under testing both in preclinical studies 4, 5, 6 and clinical trials, especially for the treatment of gliomas and prostate cancer 7. This method delivers thermal energy to the target region exploiting magnetic nanoparticles (NPs) as mediators of heating exchange and exposing the tumor to an alternating magnetic field (AMF). When magnetic NPs are injected in the tumor, and an AMF is applied, tumor cells may reach the temperature of 42-46 °C. Such a temperature increase is due to the generation of heat from magnetic NPs. Magnetic energy is indeed dissipated into heat via two different mechanisms depending on the size of the magnetic domain of the NPs. For a multi-magnetic domains NP, the heating effect is due to hysteresis losses. For a single-domain NP, it is due to the Brownian-Nèel relaxation mechanism. It involves the rapid rotation (reorientation) of the magnetic moments within the domain of the nanoparticles in the case of Nèel relaxation and physical rotation of nanoparticle within the fluid in the case of Brownian relaxation process in the presence of AMF resulting in an extremely selective thermal ablation of the tumor 8. The increase of temperature causes the activation of various mechanisms inside the cell such as unfolding and denaturation of proteins, necrosis, aggregation, apoptosis, and immune response 9, promoted by the activation of heat shock proteins and by the polarization of tumor-associated macrophages gathered by the inflammatory response of the surroundings 10.

In the last years, numerous research groups studied magnetic NPs, such as iron oxides, and ferrous fluids that could play a substantial role in treating tumors by MFH 11, 12, 13, 14, 15, 16. A widely recognized limitation of this technique is represented by the risk of overheating tumor tissue that may jeopardize surrounding healthy tissues 17.

This work aims to test an innovative, self-regulating temperature nanomaterial synthesized in the form of nanoparticles, hereafter denoted as M48. M48 belongs to a class of innovative nanomaterials developed by MBN Nanomaterialia S.p.A. (Treviso, Italy) that show self-regulating temperature 18. The self-regulating heating effect consists of a stable temperature increase of the magnetic nanomaterial when exposed to AMF up to a maximum value (T_SR_, Self-Regulating Temperature) below the Curie Temperature (T_c_). This effect provides an intrinsic control for induction heating and exploits an efficient energy conversion - from electromagnetic energy to heat - regulated by a sharp magnetic transition. The values of T_c_ can be modulated by adjusting the composition of the nanomaterial itself. The Curie Temperature is observed in correspondence to a second-order magnetic transition threshold between ferromagnetism and paramagnetism. Above T_c_, the magnetization is negligible and consequently, magnetic losses do not occur, switching off the heating mechanisms under AMF. The overheating phenomenon and the related dangerous effects on the healthy surrounding tissues can therefore be avoided thanks to the self-regulating properties of the maximum temperature level. Such properties are of paramount utility when the nanomaterial is integrated into biological matrices.

Moreover, thanks to its magnetic properties, M48 is a promising contrast agent for Magnetic Resonance Imaging (MRI) allowing in principle for simple monitoring of hyperthermia treatment and reliable control of local temperature distribution in the treated tissue. Indeed, considerable interest has been recently devoted to temperature-sensitive contrast agents for guiding thermal therapies 17, 19. However, safer and more reliable compounds for *in vivo* theranostic methods with low toxicity and tunable temperature are highly desirable.

According to the American Cancer Society, breast cancer is the second most common cancer worldwide after lung cancer, the fifth most common cause of cancer death and the leading cause of cancer death in women. Current therapeutic approaches include surgery followed by chemotherapy/radiotherapy, an invasive and debilitating approach. Novel therapy methods, minimally invasive, and devoid of negative impacts, are needed to reduce mortality rates in breast cancer patients. In this respect, MFH has been proposed as an innovative approach to breast cancer treatment 20.

In this paper, M48 was tested both *in vitro* and *in vivo* in breast cancer cells and experimental model as an efficient MFH heat mediator. An organic coating was applied to the prepared iron-based nanomaterials to guarantee biocompatibility and stability in physiological fluids. The capability to act as temperature-sensitive contrast agent for MRI was also demonstrated *in vitro*.

## Methods

### Synthesis of the iron-based NPs

M48 is synthesized by MBN Nanomaterialia S.p.A (Treviso, Italy) using a proprietary mechanochemical process 18. Briefly, M48 was prepared from MgO, Fe_2_O_3_ and TiO_2_ powders in proportion 2:4:1, performing High Energy Ball Milling 21,22 in stainless steel equipment for 6h followed by thermal treatment in air at 1200°C for 4h. The resulting powder is finely ground by a second ball milling step of 1h, then homogeneously dispersed in propan-2-ol and processed by ultrasonication to promote de-aggregation. Centrifugal classification allowed us to extract smaller particles < 200 nm. The detailed processing parameters are courtesy of MBN.

### Structural characterization

For phase analysis, an X-Ray Powder Diffractometer (Thermo ARLX'TRA) equipped with a Cu-anode X-ray source with a Peltier Si (Li) cooled solid state detector was used. The XRPD patterns were collected with a scan rate of 0.04°/s, with a measurement time of 1.0 s/step. The samples were prepared by careful homogenization in a mortar with few drops of ethanol. After evaporation of the solvent, the sample was deposited on a low background sample stage.

### Morphological analysis

The size and morphology of M48 were investigated using Transmission Electron Microscopy (TEM, FEI TECNAI G2). For TEM analysis M48 was added to the copper grid and desiccated for one day before TEM imaging.

### Coating of the iron-based NPs

The iron-based NPs were coated with citrate moieties (using Trisodium Citrate dihydrate 99% Alfa Aesar as reagent) and then with glucose (using D-(+)-Glucose SigmaUltra 99.5% Sigma as reagent). For the coating procedure, a microwave assisted synthesis was exploited to significantly reduce the reaction time. One mL of M48 suspension (concentration of 9.6 mg/mL) and 3.0 mL of sodium citrate solution (concentration of 1 M) were added in a 10 mL glass vial for the microwave assisted reaction, using an Anton Paar Monowave 400 microwave reactor. The heat treatment followed a general literature protocol with some optimizations 23 (heat treatment at 90°C for 7 min). The obtained nanoparticles were collected by centrifugation at 8000 rpm for 5 min. The whole procedure was repeated for the glucose capping (concentration of the glucose starting solution of 1M). At the end of the coating procedure, the NPs suspension was dialyzed overnight (Spectra/Por 3 Dialysis Membrane Standard RC Tubing MWCO: 3.5 kD). Hereafter, the citrate and glucose capped NPs will be referred to as G-M48.

### Dynamic Light Scattering measurements

The hydrodynamic size and the zeta potential for G-M48 were determined with a Malvern Zetasizer Nano instrument. The sample was prepared diluting G-M48 water suspension with water (1:5 in volume) in a proper plastic cuvette.

### Infrared Spectroscopy

The organic capping on the M48 surface was analyzed by using FTIR spectroscopy, with a JASCO FT/IR-660 plus. The sample was prepared by dispersing G-M48 in a KBr pellet (3 mg of NPs in 100 mg of KBr).

### Cytotoxicity assay

The cytotoxicity of G-M48 was evaluated in two cell lines: HeLa and MDA-MB-231 cells, cervix and breast cancer cell line respectively (purchased by ATCC Manassas, VA). Cells were cultured in Dulbecco's Minimum Essential Medium (DMEM) with 10% of Fetal Bovine Serum (FBS), 1% of a mix of penicillin/streptomycin 1:1 and 1% of L-glutamine 200 mM, seeded onto 96-well plates (2500 cells/well) and incubated at 37°C in humidified air with 5% CO_2_ for 24h. After 24 h, the medium was replaced with fresh medium containing 10, 50, 100, 150 ug/ml of G-M48.

The MTT (3-(4,5-dimethylthiazol-2-yl)-2,5-diphenyltetrazolium bromide cytotoxicity assay was performed after 2, 24, and 48 h of incubation: 100 µl of MTT (at 5 mg/ml concentration, purchased from Sigma, Italy) were added to each well and incubated for additional 4 h (37°C, 5% CO_2_). Formazan crystals were dissolved in 100 μl of DMSO, and the absorbance was read at a wavelength of 570 nm using a microplate reader (HTX Microplate Reader BioTek Instruments, Winooski, VT, USA). Four measurements of optical density (OD) were recorded for each sample, and cell viability (%) was calculated with the following equation: CV% = (ODsample/ODcontrol) × 100.

### Nanoparticles Internalization in cells: TEM analysis

HeLa and MDA-MB-231 cells were grown as monolayers on glass coverslips, treated with 150 μg/ml of G-M48 for 2, 24 and 48 h at 37 °C, and then fixed with 2.5% (v/v) glutaraldehyde and 2% (v/v) paraformaldehyde in 0.1 M phosphate buffer, pH 7.4, at 4 °C for 1 h. Afterward, cells were post-fixed with 1% osmium tetroxide and 1.5% potassium ferrocyanide for 1 h, dehydrated with acetone and embedded in Epon resin. Ultrathin sections were stained with lead citrate for 2 min and observed in a TECNAI G2 transmission electron microscope (FEI Company Italia Srl, Milan, Italy) operating at 80 kV and equipped with a Megaview III camera for digital image acquisition.

### Magnetic Fluid Hyperthermia

MFH was performed by using a Nanotherics MagneTherm system (Warrington, United Kingdom) in water. The heat dissipation value strictly depends on the frequency and amplitude of the applied alternating magnetic field (AMF). The transformation of magnetic energy into thermal energy mediated by magnetic nanoparticles in the presence of an external AMF is quantified from the value of specific absorption rate (SAR). The SAR value of nanoparticles in solution is calculated by using the following equation:


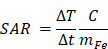


Where C is the specific heat capacity of the solvent per g, 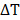 is the temperature variation in time, m_Fe_ is the mass of iron in the compound per g 21, 24.

The AMF apparatus yields a maximum magnetic field intensity of 23 kA/m (≈29 mT). A multichannel thermometer equipped with optical fiber probes (FOTEMP4, Optocon AG, Germany) was used to assess temperature variation within the sample every 10.0 s. For MFH characterization of G-M48, in order to satisfy the Hergt criterion (less rigid than the previously published Brezovich one) for the clinical translatability of the compound, a frequency *f* = 473.1 kHz (0.473 MHz) was coupled with a field strength *H* = 8.75 kA/m 25, 26, 27, 28, 29. These parameters were tested as the most efficient and clinically safe among the ones provided by the Magnetherm device, providing a coupled H*f = 4.1 10^9^ Am^-1^s^-1^ (between 0.110 and 0.970 MHz and between 1.0 and 23 kA/m ).

Thermograms of G-M48 were acquired for different concentrations (2.5, 3.75, 5, 7.5 and 10 mg/mL). The multichannel thermometer was placed inside the sample during the 20 min acquisition time. The time window of 20 min for the effective treatment was chosen considering preliminary results and experimental conditions suitable for possible translatability to the clinics. Preliminary results showed that about 15 min were necessary to reach the temperature plateau and therefore the total treatment time amounted to about 35 min (MFH protocol).

To maintain cell cultures at 37°C a homemade device constituted by a closed box with a thermostated air stream was used.

To avoid contamination of cell cultures, the temperature was calibrated before the measurement in a disposable sample for each single acquisition. For MTT assay, cells were plated on 96 well plates. Cells were incubated for 24 hours with 150 µg/mL of G-M48 and treated for 35 min with MFH protocol. Untreated cells, untreated cells subjected to MFH and cells treated with 150 µg/mL of G-M48 but not exposed to MFH were used as controls. Twenty-four hours after MFH treatment, MTT cytotoxicity assay and Hematoxylin and Eosin (H&E) staining were performed. For H&E, after the incubation time, cells were fixed with 4% buffered formalin solution for 15 min, rinsed with PBS and then stained with Mayer's hematoxylin (nucleus) and eosin (cytoplasm). Six images of each sample were taken at 10X using a BX-URA2 Olympus microscope (Olympus Optical, GMBH, Hamburg, Germany) equipped with a digital camera. MDA-MB-231 cells viability was evaluated after one, two, three and four hyperthermia treatments and normalized to that of the respective control cells.

### Characterization of G-M48 as temperature sensitive MRI contrast agent

MRI was performed using a Bruker Biospin 7T, 72 mm bore (Bruker Biospin, Ettlingen, Germany) scanner to evaluate the *in vitro* transversal relaxation rate (r_2_ coefficient), the switching contrast ratio property and the *in vivo* biodistribution of G-M48.

The transversal relaxation times and the switching contrast ratio property were measured using a standard Spin-Echo Multi-Echo sequence with the following parameters: TR = 2000 ms, TE = from 6.5 to 170.43 ms, FOV = 55 x 55 mm, matrix size = 128 x 128, slice thickness = 1 mm, number of echoes = 25. Transversal relaxation rates (1/T_2_) were plotted as a function of the Fe concentration and r_2_ relaxivity was obtained by the slope of the fitting straight line. To characterize the switching contrast ratio property, a magnetic field compatible system for the control of temperature, from 5°C up to 80 °C, was designed. A phantom was prepared with a capillary filled with G-M48 (1.9 mg/ml) solution immersed in water and settled inside the temperature-controlled box. To completely avoid the thermal stretching of the box itself, the temperature was slowly increased by 5°C every 15 min starting from 25°C.

To unveil the biodistribution of G-M48 *in vivo*, Balb/c, male mice (n = 3, 6-8 weeks old, Envigo) were used. Animals were anesthetized with gas anesthesia (a mixture of O_2_ and air containing 1-1.5% of isofluorane), placed in a heated animal bed and inserted in a 7.2 cm internal diameter bird-cage coil. G-M48 was injected at a dosage of 2 mg Fe/kg. T_2_-weighted images of the mice body were acquired using a Rapid Acquisition with Relaxation Enhancement (RARE) sequence with the following parameters: FOV = 60 x 40 mm, MTX = 256 x 256, slice thickness = 1 mm, TE = 33 ms and TR = 2.500 ms. The images were acquired before, 40 min, 24 and 72 h after G-M48 injection.

### Characterization of G-M48 nanoparticles as hyperthermia mediators *in vivo*

N = 30 nude homozygote female mice (Harlan Laboratories, Udine, Italy) were maintained under standard environmental conditions (temperature, humidity, and 12 h/12 h light/dark cycle, with water and food ad libitum) and veterinarian control in the animal facility of the University of Verona. Animal experiments were conducted following Italian law (D.L. 4 March 2014 no. 26) and the European Union normative (2010/63/EU).

Two million MDA-MB-231 cells (human breast adenocarcinoma) were injected subcutaneously in the flank of nude mice (n = 27). Starting from 15 days after cells inoculation, the size of the tumor mass was measured every three days by MRI. Three mice did not develop tumor and were excluded from the study. Twenty-one days post tumor implantation (when tumor volume reached about 200 µl) mice were divided into three groups according to the treatment. Animals in the first group (n = 8, CTRL) received saline intratumorally. Animals in the second group (n = 8, G-M48) received G-M48 [1.2mg Fe/mL]. Animals in the third group (n = 8, G-M48 + MFH) received G-M48 [1.2mg Fe/mL] and were exposed to four MFH cycles immediately, 24h, 72h and 96h after G-M48 injection. In order to improve the homogeneity of the distribution of G-M48 within the tumor tissue, the total injected volume (100 µl) was divided in 5 aliquots and injected in different areas of the tumor. Each MFH treatment consisted of 35 min application (in agreement to the MFH protocol adopted for the *in vitro* study) of an oscillating magnetic field with a maximum intensity of 23 kA/m (≈29 mT) and a frequency of 473 kHz. In particular, it was chosen the same field strength and frequency used for the *in vitro* studies (*H* = 8.75 kA/m and *f* = 473.1 kHz). During MFH treatments, animals were maintained at 37°C using a homemade device constituted by a closed box with a thermostated air stream.

All mice were monitored by MRI before (day 1), 3 days (day 3) and 6 days (day 6) after the injection of G-M48 to monitor the efficacy of treatments. T_2_ weighted images were acquired in the axial plane using a RARE 3D sequence with TR = 1200 ms, TE_eff_ = 47.5 ms, NEX = 1, field of view = 25 × 25 × 25 cm^3^, matrix size (MTX) = 256 × 128 × 32, slice thickness = 0.8 mm, flip angle = 90°, RARE factor = 16. To measure the volume of tumors, MR images were processed with Paravision software and a custom-made MATLAB (MathWorks, Natick, USA) script. The tumor area was manually selected slice by slice and tumor volume was calculated as 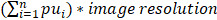, where *pu_i_* represents the number of pixels manually selected. The percentage increase in tumor volume was calculated at each time point as 100* (V_t_-V_0_)/V_0_, where V_t_ is the tumor volume at time t and V_0_ is the initial volume.

### Histology

Mice were sacrificed and tumor, liver, kidneys were dissected out, washed with PBS 0.1 M and fixed in 10% formalin for 4 hours. Tissues were embedded in paraffin, cut in 5 µm thick sections with a microtome and dried at 37°C for 24 h. To evaluate the presence of iron in the tissue, Prussian Blue (PB) staining was performed: sections were incubated with PB solution (5% hydrochloric acid and 5% potassium ferrocyanide) for 40 min and counterstained with nuclear fast red (Bioptica) for 10 min. Sections were examined under a light microscope (Olympus BXS1) equipped with a charge-coupled device camera. Finally, to evaluate tissue damage in tumor tissue, sections were stained with Hematoxylin and Eosin (H&E).

### Statistical Analysis

Statistical analysis was performed using Prism software (8.1.1, GraphPad Inc., La Jolla, CA, USA) and confirmed by a custom script for statistical analysis realized in MATLAB (Mathworks, Natick, MA, USA). All the data are expressed as mean ± standard deviation of the mean (Mean ± SEM). Statistically significant differences were evaluated by TWO-way ANOVA with multiple comparisons using the Tukey-Kramer method. Differences were considered statistically significant when the p-value < 0.05.

## Results and Discussion

### Morphological and structural characterization

**Figure 1:**
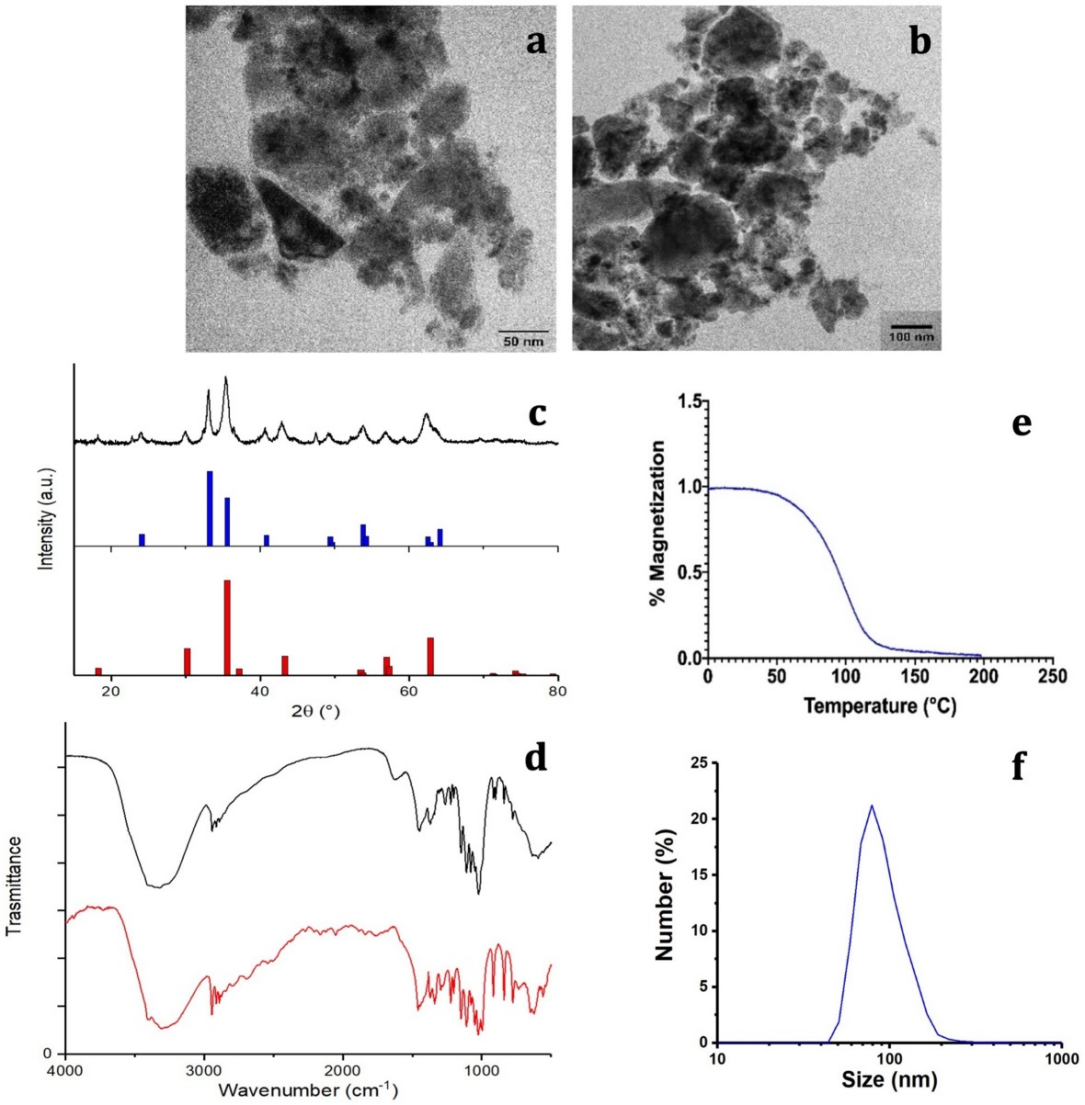
Characterization of M48 and G-M48 nanoparticles. a, b) TEM images of M48; c) XRPD pattern of M48: black line, experimental data; red pattern, Fe3O4 (magnetite, COD n.9002319), blue pattern, alpha-Fe2O3 (hematite, COD n.9000139); d) FTIR spectrum of G-M48 (black line); FTIR spectrum of glucose (red line), e) M48 magnetization vs temperature; f) Hydrodynamic diameter (by Dynamic Light Scattering) of G-M48.

TEM allowed investigation of M48 morphology. TEM images, reported in Figure 1 (a,b), reveal that M48 nanoparticles have a large size dispersion and are present as single nanoparticles or aggregates with size ranging from around 10 nm to more than 100 nm. A decreased particle size distribution could positively affect the *in vivo* application of M48 nanoparticles. For example, reduction of size could affect the biodistribution with decreased liver uptake that is desirable in the prospective of systemic administration. Decreasing particles size could however also affect their magnetic properties. When the size of the NPs is of the order of the size of magnetic domain, there will be a switch to superparamagnetic single domain NPs with no transition. The optimization of size distribution of the magnetic core of the NPs is a very important future development of our work and will be the objective of further investigations.

The composition (both phase and structural characterization) of M48 was investigated by the XRPD technique. The measured XRPD pattern obtained for the M48 sample is shown in Figure 1 (c) (black line). A detailed analysis of the experimental XRPD pattern revealed that the M48 sample is composed by more than one iron oxide compounds. This behavior can be observed in Figure 1 (c), where the XRPD patterns of 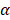-Fe_2_O_3_ (hematite, Crystallography Open Database (COD) n. 9000139) and Fe_3_O_4_ (magnetite, COD n. 9002319) are shown in blue and red patterns, respectively. From an EDX analysis of the M48 sample, also Mg and Ti metals were found, and a relative molar ratio Fe:Mg:Ti = 1:1:0.3 was evaluated. This evidence indicates that other iron-containing compounds, as magnesium ferrites or iron titanates, that have XRPD patterns compatible with the experimental one, can be present in the sample.

### Coating of M48 nanoparticles

To use the sample in biological systems, M48 NPs were surface coated with glucose molecules and citrate ions moieties. The idea of using two hydrophilic coating agents was pursued to provide high colloidal stability and good biocompatibility of the NPs.

As described in the experimental section, the NPs were capped with citrate ions and glucose moieties. Citrate capping was chosen as this capping enhances the stability of inorganic NPs in water 30. On the other hand, glucose coating was chosen considering the pronounced glucose greed of tumor cells. Indeed, malignant tumors exhibit a high rate of glucose uptake compared to normal cells. Glucose molecules are internalized via GLUT transporters, highly expressed on the cancer cell surface 31. For this reason, glucose coating is an effective way to facilitate the entry of NPs into tumor cells.

The surface coating was characterized using FTIR spectroscopy. Figure 1 (d) shows the FTIR spectrum of the citrate and glucose coated M48 sample (hereafter denoted G-M48) compared with the glucose molecule's FTIR spectrum. Several features in the FTIR spectrum of G-M48 are due to the glucose molecule, confirming that glucose is present on the nanoparticles surface. On the other hand, it was impossible to distinguish any characteristic band of the citrate ions, most probably due to a higher amount of glucose than the citrate ions on the nanoparticle surface. Figure 1 (d) shows one broadband around 3300 cm^-1^ due to -OH stretching of glucose or citrate ions and a narrow band around 2900 cm^-1^ due to -CH stretching of the organic backbone. In the region between 1600 and 500 cm^-1^, many vibrations due to the glucose molecular skeleton are observed, in substantial agreement with those observed for the FTIR spectrum of the glucose molecule (red line in Figure 1(d)), in the fingerprint region. Besides, an absorption band around 570 cm^-1^ can be assigned to a Fe-O vibration of the inorganic phase.

### Colloidal and magnetic properties of water dispersion of G-M48

Dynamic Light Scattering (DLS) was exploited to investigate the colloidal stability of water dispersion of G-M48 through the measurement of the zeta potential. The obtained zeta potential value of -23.2 ± 10.7 mV, reported in Figure S1, confirms good colloidal stability 32.

The thermomagnetic analysis was performed on dry M48 nanoparticles, measuring magnetic susceptibility variation with temperature, on-heating between -85 and 200 °C, and on-cooling down to 40°C, in inert Argon atmosphere, applying a 10 Oe magnetic field at 502 Hz. A Curie temperature (T_c_) of 95 °C was determined as the temperature of susceptibility at half height of the curves normalized at the maximum peak before susceptibility drop. Indeed, thanks to their innovative design and synthesis approach, M48 at low temperature is in an ordered state with low entropy, a so-called ferromagnetic state, and can undergo a second-order phase transition into a paramagnetic state at a specific temperature T_c_ (T_c_ = 95°C).

### Cytotoxicity and cell internalization

**Figure 2:**
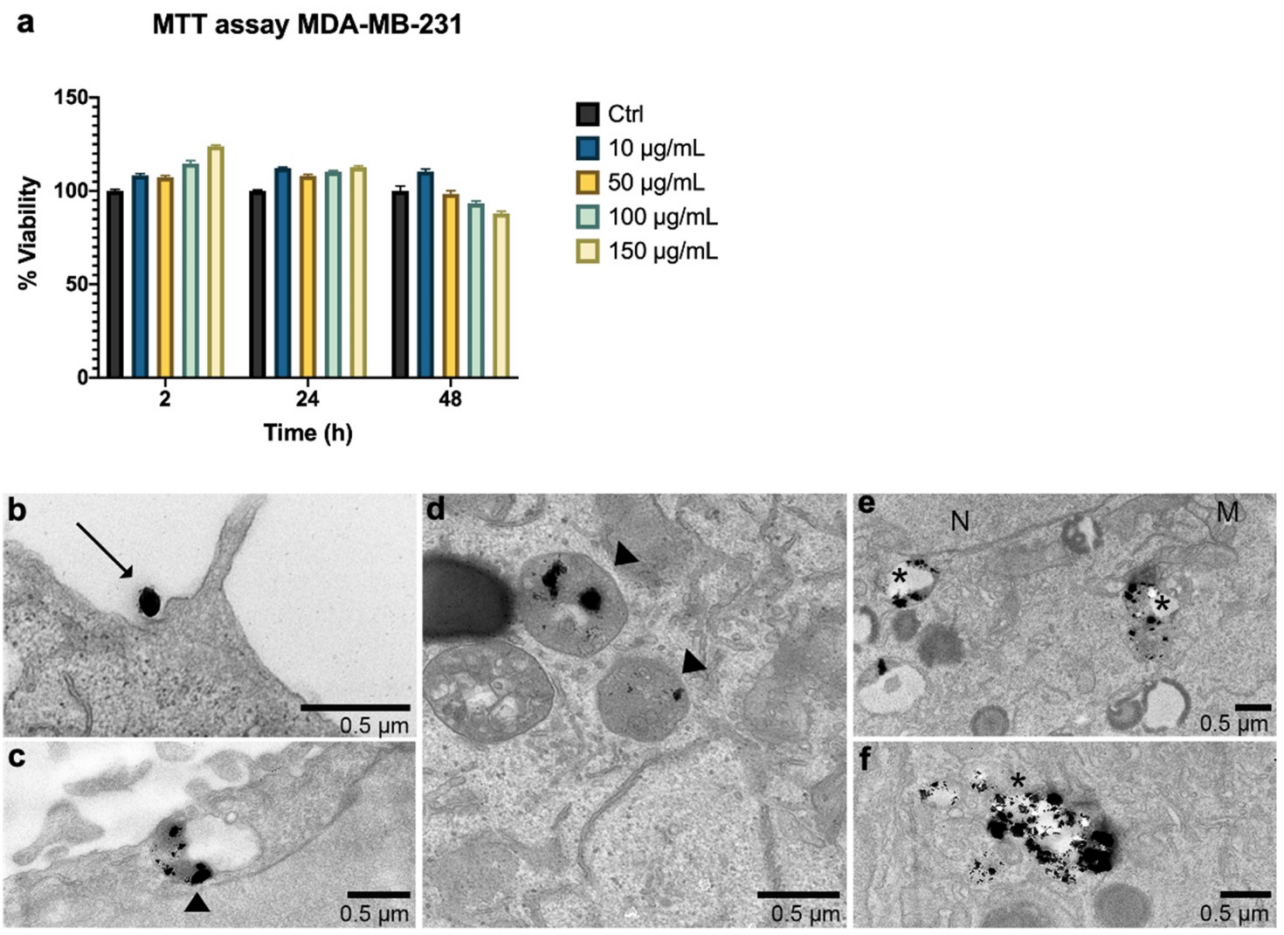
*In vitro* viability of MDA-MB-231 cells and G-M48 internalization at TEM. a) MTT assay in MDA-MB-231 cells shows that G-M48 is safe up to a concentration of 150 μg/mL and up to 48 h of incubation time; the error bar represents SEM over six replicates. Transmission electron microscopy images of G-M48 uptake and distribution in MDA-MB-231 cells. b) MDA-MB-231 cells internalize G-M48 via endocytosis (arrow). c-f) G-M48 nanoparticles are compartmentalized into endosomes (arrowheads in c,d) distributed in the cytoplasm, as well as in residual bodies (asterisks in e,f). MDA-MB-231 cells show well-structured mitochondria (M) and endoplasmic reticulum cisternae, demonstrating no cell damage. N: cell nucleus.

An important feature for the biomedical application of nanoparticles in MFH is the absence of cytotoxicity when used without AMF. Cell viability was therefore assessed after 2, 24, and 48h of incubation with G-M48 in MDA-MB-231 (Figure 2 (a)) and HeLa cells (results in supplementary information). MTT assay reveals that G-M48 is safe up to the concentration of 150 μg/mL for long incubation times (48 h, Figure 2 (a)). The electron-dense chemical composition of G-M48 allowed to unveil the intracellular distribution and the internalization process of the nanoparticles in MDA-MB-231 cells (Figure 2 (b-f)) at different incubation times. In the cytoplasm, G-M48 nanoparticles were always found enclosed into endosomes (Figure 2 (c-d), arrowheads) and, following the lytic pathways, secondary lysosomes/residual bodies (Figure 2 (e), 2 (f), asterisks). G-M48 nanoparticles were thus always entrapped in vesicular structures and never free in the cytoplasm or in contact with any organelle, confirming that endocytosis can be identified as the primary internalization process in MDA-MB-231 cells. Mitochondria, endoplasmic reticulum and Golgi complexes preserved their morphology, confirming the absence of cell damage or death up to 48 h of incubation time.

### *In vitro* Magnetic Fluid Hyperthermia characterization

**Figure 3:**
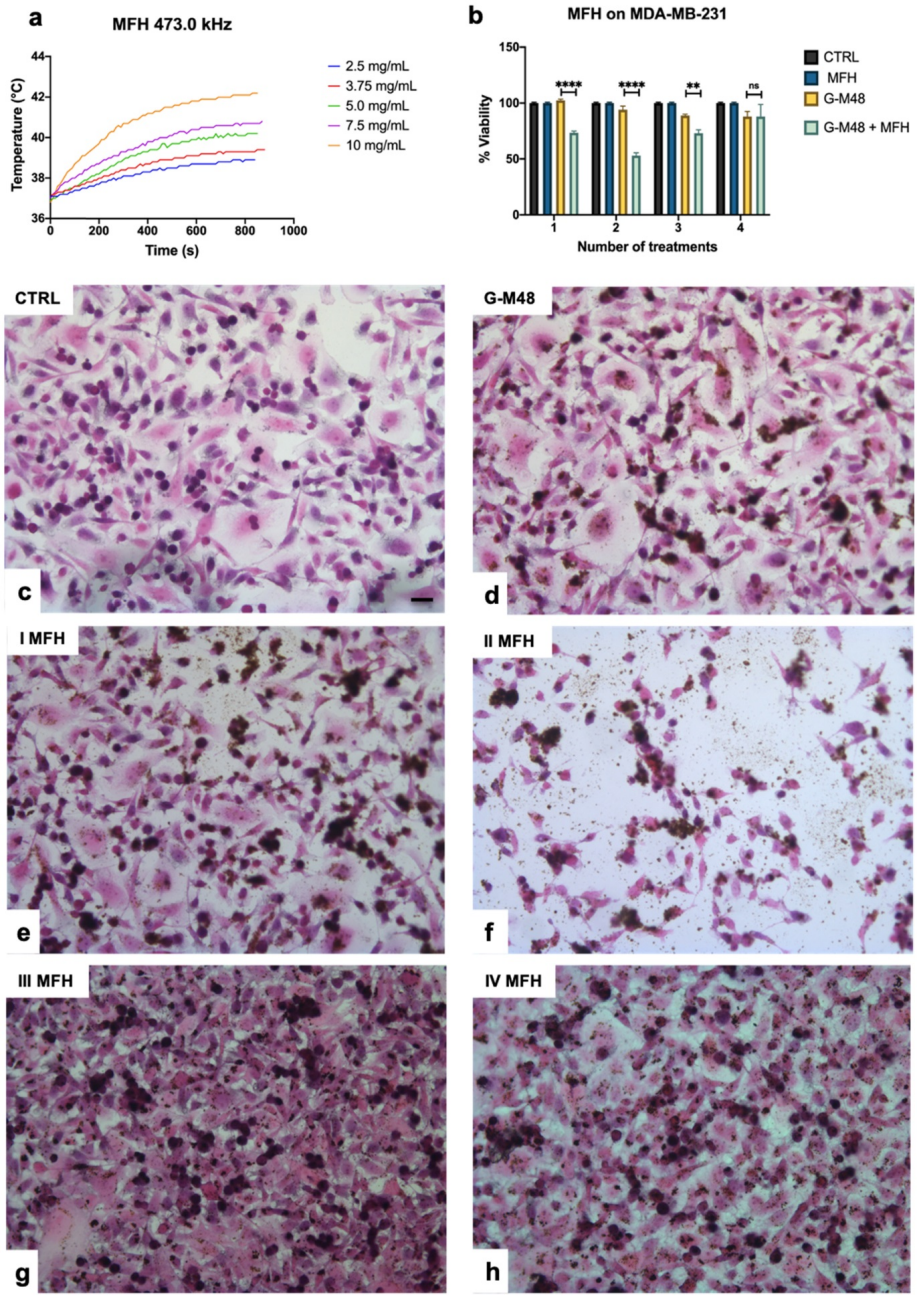
Viability of MDA-MB-231 after MFH treatments and related histologies of the different groups. a) Thermograms were collected in water solutions at different concentrations of G-M48 suspension; b) MTT assay on MDA-MB-231 cells after multiple MFH treatments (each every 24 h). (c-h) Light microscopy images of H&E stained MDA-MB-231 cells: c) control cells, d) cells incubated with G-M48 without AMF application, e) f), g), h) Cells incubated with G-M48 and subjected to I, II, III, IV MFH treatments and observed 24 hours after the treatment. Scale bar: 50 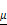
m.

Figure 3 (a) reports the thermograms up to the plateau collected in aqueous suspensions of G-M48 at different concentrations. As expected, an increase in NPs concentration (and so in the iron content) increases temperature variation. Thermograms were collected starting from a specific bulk temperature, in this case, 37.0 °C. This starting condition was chosen to simulate the condition of *in vivo* experiments. As reported in Figure 3 (a), the most concentrated sample reveals a promising increase of temperature of 5.5 ± 0.5 °C that paves the way to more exciting results *in vitro* and *in vivo*. For each sample the temperature plateau was reached approximately after 15 minutes. The SAR of G-M48 was determined in aqueous solution from the acquired thermograms, and a value of 47.0 ± 8.0 Wg^-1^ was obtained. SAR depends on various parameters such as size, shape, and magnetic properties of nanoparticles, frequency, and intensity of the applied external AMF 33. The result agrees with typical SAR values reported in the literature for iron oxide nanoparticles (ranging from 20 to 400 Wg^-1^) 33, 34.

As reported in Das et al. 2019 34, cancer cells are more sensitive to temperature enhancement than non-tumoral cells, and usually, temperature around 46 °C can induce death in tumor cells. According to literature, MFH involves cancer treatment in a temperature range of 40-46 °C, at which denaturation and aggregation of intracellular proteins induce cell death 35.

During the early days of magnetic hyperthermia applications for cancer therapy it was commonly used the Brezovich criterion in order to identify an upper limit to the magnetic field strength and the frequency applied in the treatments. This limit was calculated through the equation C = H*f (*H* is the magnetic field intensity in Am^-1^ and *f* is the frequency of the related field in s^-1^) and it is equal to 4.85 10^8^ Am^-1^s^-1^. Nowadays it is accepted another safety criterion, the so called Hergt criterion. It is a less rigid rule that was introduced to adapt the magnetic hyperthermia procedure to different geometries and shapes of the treated body. It can be summarized as C = H*f 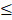 5.0 10^9^ Am^-1^s^-1^. Considering this rule, the frequency and the magnetic field strength were chosen according to the Hergt criterion with a final value of *C* = 4.1 10^9^ Am^-1^s^-1^, perfectly fitting the above condition 29.

G-M48 was tested as hyperthermia mediator on MDA-MB-231 cells at the concentration of 150 μg/mL, verified to be the highest non-toxic concentration (Figure 3 (b)). MDA-MB-231 cells were incubated with G-M48 for 24h and exposed to four cycles of AMF spaced 24h one each other.

Every effective cycle lasted 20 min (following the initial 15 minutes, necessary to reach the temperature plateau). Then, cell viability was determined 24h after the last hyperthermia treatment and normalized to the related control cells, as shown in Figure 3 (b). The percentage of cell viability was significantly affected by MFH treatment compared to control conditions (untreated cells, untreated cells subjected to MFH, and cells treated only with G-M48 but not exposed to MFH). MDA-MB-231 cells treated only with G-M48 but not exposed to MFH showed a percentage of viability of 95.3 ± 4.3%, while cells treated with G-M48 and exposed to MFH showed a percentage of viability of 73.0 ± 2.5% and 52.0 ± 4.3% after the first and the second hyperthermia treatment, respectively. After the third and the fourth treatment, the viability recovers up to 73.0 ± 5.2% and 88.0 ± 18.0%.

Histological examination of MDA-MB-231 cells stained with H&E confirmed the results obtained by MTT (Figure 3 (c-h)). Cells incubated with G-M48 and subjected to AMF demonstrated a more pronounced cell death effect after the first (e) and the second (f) MFH treatment in comparison with control cells (c) or to cells incubated with G-M48 without AMF application (d). In agreement with the MTT data, after the third and fourth treatment, cell viability recovered. The observed recovery effect can be partially attributed to cell replication and consequent G-M48 dilution. Moreover, it is well-known that hyperthermia stress induces the synthesis of a class of proteins named 'heat shock proteins' (Hsps) 36, which may induce thermoresistance with a consequent decrease of the efficacy of the last two treatments 37, 38, 39.

### Characterization of G-M48 as MRI temperature sensitive contrast agent

**Figure 4:**
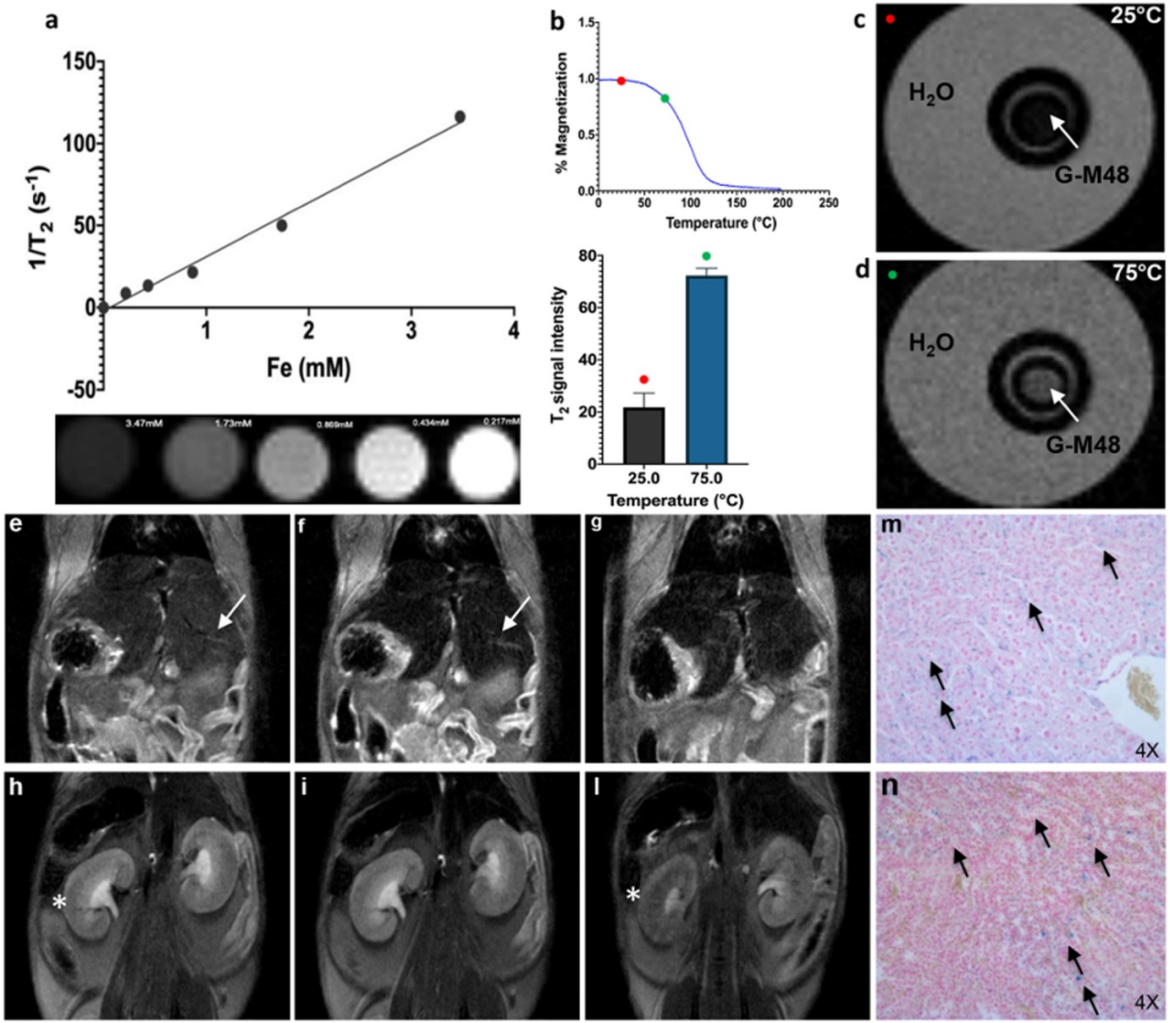
Characterization of G-M48 as temperature sensitive MRI contrast agents. *In vivo* biodistribution and clearance verified by MRI and histological examination. a) The plot of transversal relaxation rate versus Fe concentration in water dispersed G-M48 phantoms, at 25°C. MRI images of phantoms cross section are also reported. b) SNR of G-M48 water suspension at 25 and 75°C and related magnetization curve of M48. The T_2_ signal variation has been observed in the related histogram. c-d) MRI images of a phantom constituted by a capillary filled with G-M48 (white arrow) and placed inside a thermostated box at 25°C and at 75 °C. MR images of a representative animal acquired using a T_2_-weighted sequence before (e, h), 40 min (f, i) and 72 hours (g, l) after the injection of G-M48. The liver and the kidney are indicated by arrows in Figure 4 (e-h), respectively. After 40 min the signal intensity (S.I) in the liver dropped by 32.6% (Figure 4 (f) arrow). In the kidneys, no appreciable loss of signal could be detected 40 min post-injection, while at 72 hours the S.I decreased by 45 % (Figure 4 (l)). Prussian Blue staining of liver and kidneys 72h after administration of G-M48 (Figure 4 (m, n), respectively).

The transversal (spin-spin) relaxation time of water dispersion containing G-M48 was measured at 7T in a series of phantoms with variable iron concentration (from 0.21mM to 3.47 mM). Values of 1/T_2_ vs. Fe concentration were interpolated using a straight line whose slope determines the transversal relaxivity of the NPs under investigation (Figure 4 (a)). As shown in the same figure, the T_2_-weighted MR images of G-M48 phantoms tend to become darker with increasing iron concentration showing that G-M48 can effectively reduce the spin-spin relaxation time of water protons as a T_2_ contrast agent. Transversal relaxivity (r_2_) of 32.10 mM^-1^ s^-1^ was obtained, comparable to the value of a commercial iron oxide MRI contrast agent, like Endorem 35, 36 confirming the potential of G-M48 as a contrast agent for MRI.

As reported in Figure 1 (c), the magnetization of M48 strongly depends on temperature through a second-order magnetic phase transition occurring at 95°C (T_c_). Therefore, a relevant decrease in the transversal relaxivity is expected as temperature increases. Figure 4 (c, d) shows MR images of a phantom containing a G-M48 filled capillary embedded in water acquired at 25°C (i.e., well below T_c_) and at 75°C (i.e., not far from T_c_), corresponding to the red and green dots respectively in Figure 4 (b). A substantial increase in the signal of the G-M48 filled capillary is visible. The histogram shows the quantitative signal to noise ratio (SNR) of G-M48 capillary that increases from 21.8 ± 5.4 (T = 25 °C) to 72.3 ± 2.0 (T = 75°C). This property allows G-M48 to be a temperature-sensitive contrast agent. The SNR increase could be more relevant if observed at 95°C, but the available experimental apparatus could not be used at temperatures higher than 75 °C. However, as an iron-based contrast agent, it has to be considered the well-known issue related to the susceptibility artifacts that produces in MRI images “signal voids” in large areas surrounding the iron core. High local concentrations of NPs, as those used in MFH, may therefore destroy the signal of tumor tissue and prevent the observation of tumor anatomical details.

The currently used nanoparticle has a ferromagnetic-paramagnetic transition at 95°C, while a substantially lower transition temperature (around 42-45°C) should be necessary for safe and effective translation to the clinic. However, the present nanoparticle belongs to a class of nanomaterials whose transition temperature can be modulated by adjusting the composition of the nanomaterial itself. The present is a proof-of-concept of the usefulness of such materials as temperature sensitive contrast agent and future investigations will be devoted to preparation and characterization of nanoparticles with ferromagnetic-paramagnetic transition occurring at lower temperature.

Finally, the biodistribution and clearance of M48 nanoparticles were investigated *in vivo*. G-M48 was intravenously injected in healthy mice and monitored up to 72h. In Figure 4, MR images of a representative mouse acquired using a T_2_-weighted sequence before (e, h), 40 min (f, i), and 72 (g, l) hours after the injection of G-M48 are shown. A dosage of 2 mg Fe/kg was administered. The signal intensity (S.I) in the liver decreased by 32.6 ± 5.2% (Figure 4 (f) arrow) and by 46.8 ± 3.9% 40 min and 24 h after injection, respectively, indicating early hepatic accumulation of the nanoparticles. Seventy-two hours after G-M48 injection, the S.I in the liver dropped by 58.13±4.5% (Figure 4 (g)). In the kidney, no appreciable loss of signal could be detected 40 min post-injection (p.i.), while 72 h p.i. the S.I decreased by 45 ±7.3% as it is visible in Figure 4 (l) (asterisk). The loss of S.I in both cortex and medulla of the kidney observed 72h p.i. may indicate renal clearance of G-M48. No loss of signal was detected in the spleen.

To validate MRI results, histological examination was performed. Prussian Blue staining of liver and kidneys (see Figure 4 (m, n)) showed several blue spots homogeneously distributed in the tissues indicating the presence of iron. Of note, the tissues exhibit no relevant modifications in the parenchyma with good preservation of cellular morphology, as shown in Figure 4 (m, n) for liver and kidney, respectively, indicating that G-M48 has a good biocompatibility.

### Characterization of G-M48 as hyperthermia mediators *in vivo* in an experimental model of breast cancer

**Figure 5:**
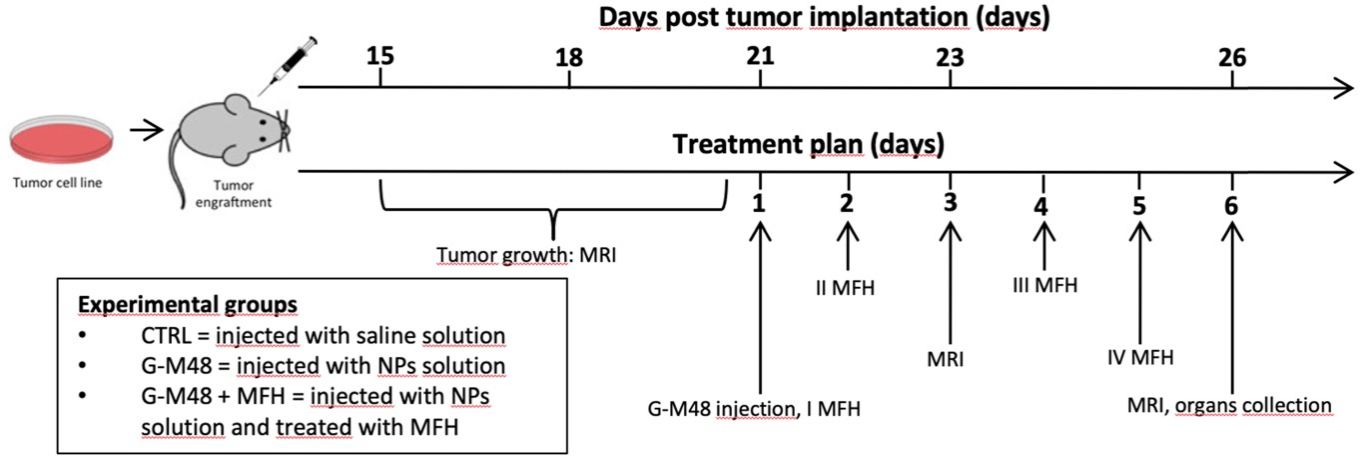
Schematic illustration of the *in vivo* experiment.

The experimental plan illustrated in Figure 5 was implemented to test the efficacy of G-M48 as an MFH mediator *in vivo*.

**Figure 6:**
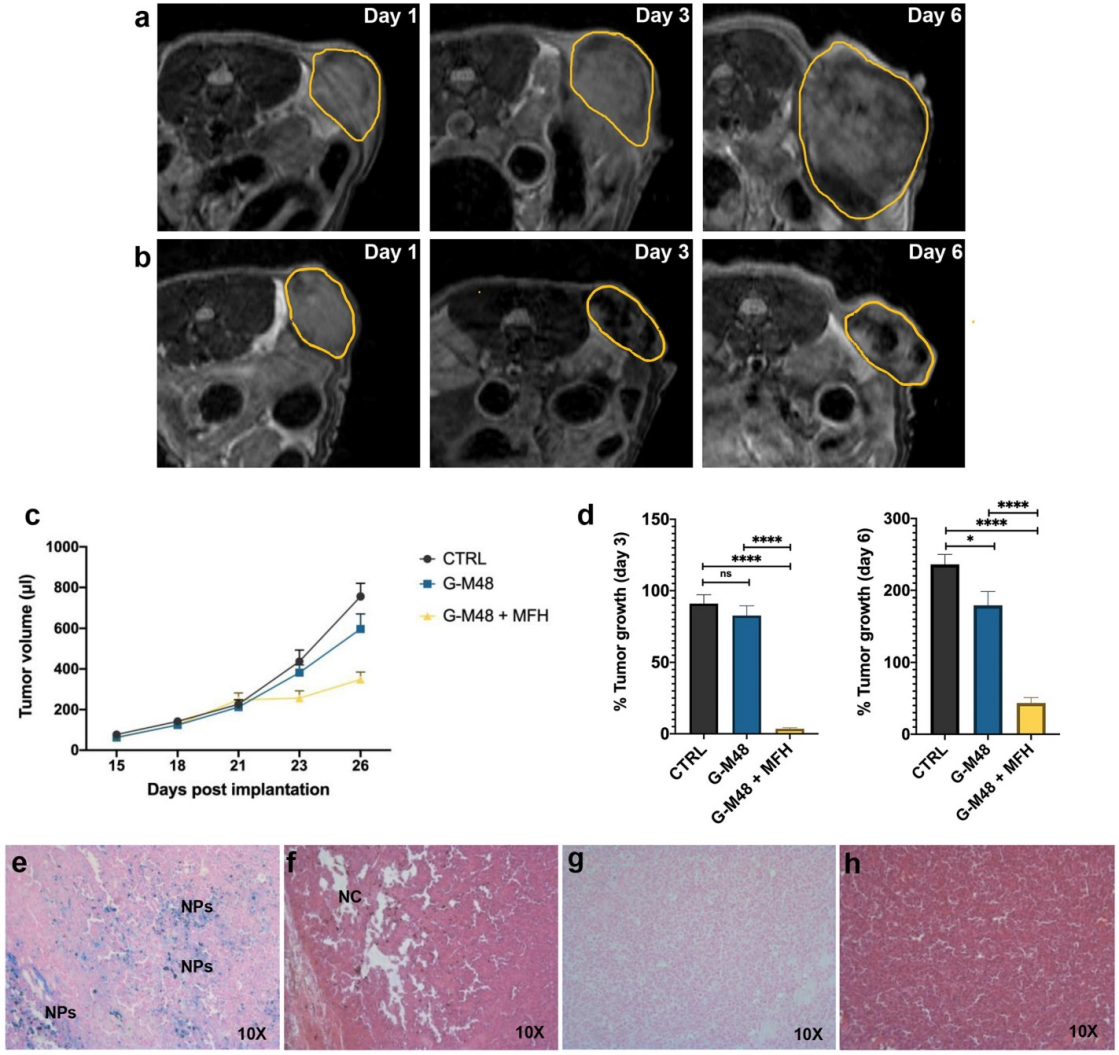
MRI monitoring of the % of tumor growth and histological examination. Representative MR images of the tumor's growth for the group administered with saline solution (a) and G-M48 + MFH (b). (c) Average tumor volume at different time points after tumor implantation (mean ± SEM). (d) % Tumor growth 3 and 6 days after the first MFH treatment (corresponding to 23 and 26 days after tumor implantation, respectively). A TWO-way ANOVA statistical test and a multiple comparison Tukey-Kramer test was used to assess the statistical significance of the % Tumor growth. (e-f) Histological slices of tumors treated with G-M48+MFH stained with Prussian Blue (e) and H&E (f). (g-h) Histological slices of tumors treated with saline and stained with Prussian Blue (g) and H&E (h).

Twenty-one days after cell implantation (tumor volume approximately 200 ul), G-M48 nanoparticles were intratumorally injected in mice. Four MFH treatments consisting of 20 min of exposure to AMF were applied (following the initial 15 minutes, necessary to reach the temperature plateau). Figure 6 (a-b) shows representative MRI images acquired in two mice belonging to the CTRL group and the G-M48 + MFH group. MRI images clearly show that tumor growth is considerably faster in the CTRL group than in the treated group. Moreover, the presence of G-M48 in the tumor tissue is detectable by MRI as signal voids, according to its ability to behave as a negative contrast agent (Figure S4, supplementary information, shows additional images). The tumor volume progression for the three experimental groups is reported in Figure 6 (c). The tumor volume does not differ among the three groups until day 21st after tumor cell implantation, which corresponds to day 1 of the treatment. After the second MFH cycle (24 hours after the first treatment), the tumor volume in G-M48 + MFH mice remains substantially lower than in control groups (saline solution and G-M48) (Figure 6 (c)). The percentage increase of the tumor volume for the experimental groups on day 3 and day 6 is shown in Figure 6 (d). Values are expressed as percentages of the initial size, measured at day 1 of the treatment plan (Figure 5). Data are reported as mean ± SEM. On day 3, while the tumor volume of group G-M48 + MFH is stable (3.45% ± 1.59) compared to the initial volume, the tumor volume of CTRL and G-M48 groups strongly increases by 91.11% ± 13.50 and 82.67% ± 16.68, respectively. At day 6, the tumor volume in the group G-M48 + MFH remains significantly smaller (43.47% ± 17.20) than either the CTRL group (236.09% ± 30.99) or the G-M48 group (179.51% ± 46.23), showing that G-M48 strongly inhibits tumor growth when applied as MFH mediator. It is noteworthy that a small difference in % tumor growth was also detected between CTRL and G-M48 groups. This may indicate marginal toxicity of the nanoparticles themselves that was not evidenced *in vitro*. It has to be considered that *in vivo* fate of injected nanoparticles could differ from *in vitro*: as apparent from MRI, upon intratumoral injection, nanoparticles accumulate heterogeneously in the tumor mass and, therefore, may give rise to local areas of very high concentration and toxicity. Future efforts will be devoted to minimizing such heterogeneous distribution in tumor tissues to optimize toxicity and efficacy.

The progress of tumor volume reported in Figure 6 (c) shows that the highest efficiency (no increase of tumor volume) is reached after the second MFH treatment in agreement with *in vitro* cell culture studies. Single heat treatment could induce irreversible protein damage and result in protein aggregation or inhibition of cellular functions. Moreover, it is well known that mild hyperthermia treatments can induce and regulate apoptosis and heat shock proteins (Hsps) expression. The tissue level effects include pH changes, alterations in perfusion, and oxygenation of the tumor micro-environment. To get more insight into cell death mechanisms and their dynamics, further studies will be needed to identify expression of different biomarkers related to necrosis and apoptosis. The effectiveness of any hyperthermia treatment greatly depends on the temperature generated at the targeted sites of action, duration of exposure, and specific cancer cells 23, 35, 42, 43.

At day 6 of the treatment, mice were sacrificed, and the tumor masses excised for histological analysis. Figure 6 (e-h) shows histological slices of tumors treated with G-M48 + MFH and CTRL. Prussian Blue staining confirmed the presence of iron inside the tumor mass (see blue spots indicated by 'NPs') in the G-M48 + MFH group (e). H&E staining revealed damaged regions characterized by a paucity of cells distributed in different portions of the tumor (Figure 6 (f)) in the G-M48 + MFH group. The saline group resulted negative for Prussian Blue staining and did not exhibit apparent modifications in the parenchyma with good preservation of cellular morphology in the core and in the peripheral area of the mass (Figure 6 (g-h)). Good preservation of tumor tissue with the absence of necrotic areas was detected in the G-M48 group whose H&E histological slices closely resembled those of the CTRL group (see Figure S5).

## Conclusion

This study characterized an innovative nanomaterial, M48, as a theranostic agent, combining diagnostic imaging with local treatment of cancerous tissues. The results show that a double shell coating process makes M48 water-soluble, biocompatible, and safe both *in vitro* and *in vivo*. T_2_ weighted MR images of G-M48 phantoms performed at 25°C and 75°C demonstrate the nanomaterial switching contrast property, opening new scenarios in MFH treatments controlled by MRI imaging. The biodistribution of G-M48 was investigated by MRI: clear and early loss of signal was detectable in the liver (40 min p.i.) while late loss of signal was detectable in kidneys (72h p.i.).

Finally, MFH experiments confirm the ability of G-M48 to act as a hyperthermia mediator both *in vitro* and *in vivo*. Results obtained *in vivo* are particularly interesting: after the first two MFH treatments, G-M48 nanoparticles strongly inhibit tumor growth (% tumor growth 3.45% ± 1.59 vs. 91.11% ± 13.50 in the CTRL group).

Overall, our results pave the way for efficient and minimally invasive cancer therapy and diagnosis by applying a single NP as a hyperthermia heating agent and MRI contrast agent with self-regulating temperature properties.

## Supplementary Material

Supplementary figures.

## Abbreviations

AMF: alternating magnetic field
COD: crystallography open database
DMEM: Dulbecco's Minimum Essential Medium
DLS: Dynamic Light Scattering
FBS: fetal bovine serum
Hsps: heat shock proteins
H&E: Hematoxylin and Eosin
MRI: magnetic resonance imaging
MFH: magnetic fluid hyperthermia
Min.: minutes
NPs: nanoparticles
p.i.: post injection
PB: Prussian Blue
RARE: rapid acquisition with relaxation enhancement
T_c_: Curie Temperature
T_SR_: self-regulating temperature
S.I.: signal intensity
SNR: signal to noise ratio
SAR: specific absorption rate
TEM: transmission electron microscopy
XRPD: X-Ray Powder Diffractometer
