# Supplementary material for: Nanoparticles exhibiting self-regulating temperature as innovative agents for Magnetic Fluid Hyperthermia
Source: Nanotheranostics. 2021 Mar 15;5(3):333–47. doi: 10.7150/ntno.55695 (PMC7961124; doi:10.7150/ntno.55695)
Supplement: Supplementary file 1 — Supplementary figures. [file ntnov05p0333s1.pdf]

Supplementary Information

Nanoparticles exhibiting self-regulating temperature as innovative agents for Magnetic Fluid Hyperthermia

Marco Gerosa[1*], Marco Dal Grande[2*], Alice Busato[3-4*], Federica Vurro[3], Barbara Cisterna[4], Enrico Forlin[5], Filippo Gherlinzoni[6], Giovanni Morana[6], Michele Gottardi[6], Paolo Matteazzi[5-6], Adolfo Speghini[2], Pasquina Marzola[3]

[1]Department of Diagnostics and Public Health, University of Verona, Piazzale L.A. Scuro, 37134 Verona, Italy
[2]Nanomaterials Research Group, Department of Biotechnology, University of Verona and INSTM, RU Verona, Strada Le Grazie 15, I-37134 Verona, Italy
[3]Department of Computer Science, University of Verona, Strada Le Grazie 15, 37134 Verona, Italy
[4]Department of Neurosciences, Biomedicine and Movement Sciences, University of Verona, Piazzale L.A. Scuro 10, 37134 Verona, Italy
[5]M.B.N. Nanomaterialia S.p.A., Via Giacomo Bortolan, 42, 31050 Carbonera Treviso, Italy
[6]Foundation for Nanotheranostics Research in Cancer therapy RNC, Treviso, Italy
Corresponding author: pasquina.marzola@univr.it

* These authors contributed equally to this work.

Key words: Magnetic Fluid Hyperthermia, Self-regulating temperature, Curie Temperature, Nanoparticles, MRI

Zeta Potential measurement of G-M48

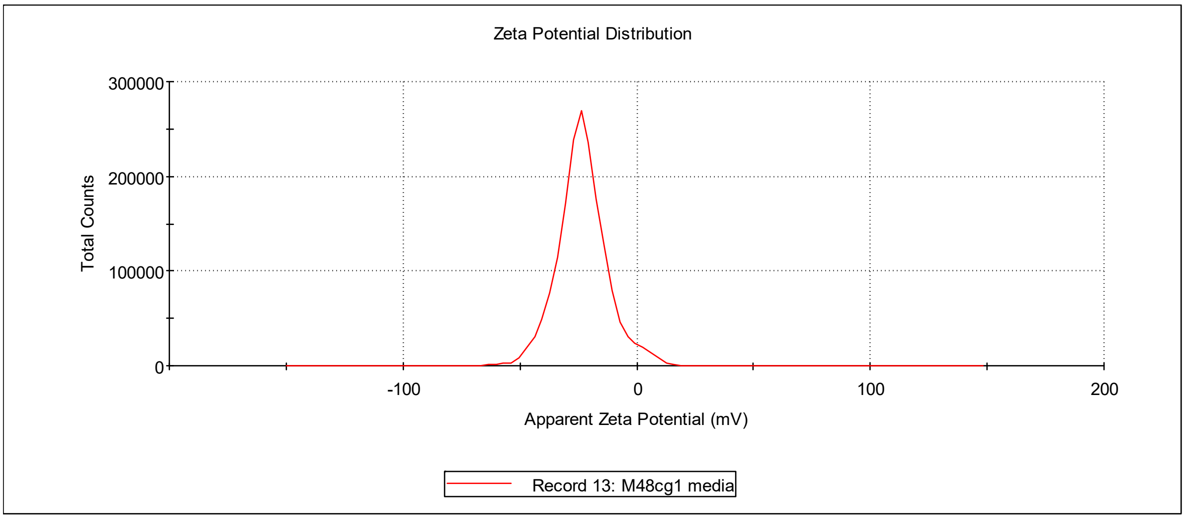

Figure S1. Zeta Potential for G-M48 nanomaterials.

Cytotoxicity in HeLa Cells

Cytotoxicity of G-M48 was evaluated on HeLa cells treated with increasing concentration of NPs (10, 50, 100 and 150 µg/ml) and incubated for 2, 24 and 48h. Results of toxicity tests are reported in

Figure S2. MTT assay reveals that G-M48 does not affect in a statistically significant manner HeLa cells viability until 48h.

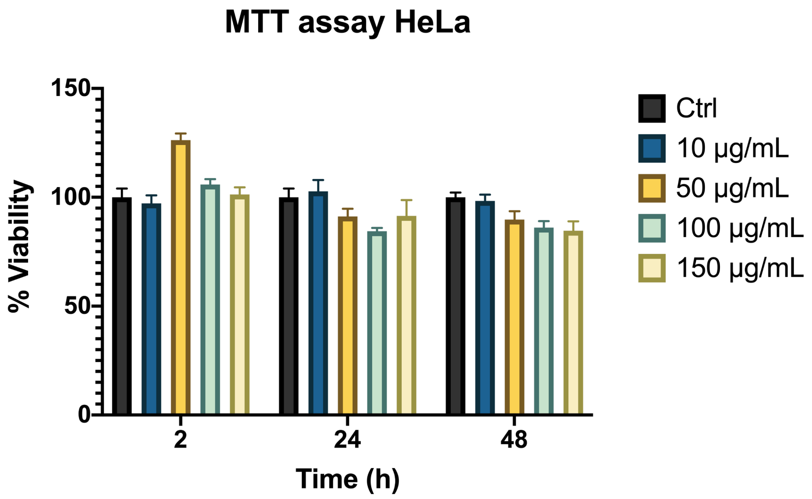

Figure S2. MTT assay in HeLa Cells incubated with G-M48 (10-150 µg/ml) for 2, 24 and 48h of internalization.

In vitro Magnetic Fluid Hyperthermia in HeLa cells

HeLa cells were incubated with G-M48 and subjected to MFH treatment for 20 minutes. Cell viability was determined by MTT assay, 24h after the hyperthermia treatment, and normalized respect to the related control cells. Different concentration of G-M48 (100, 300 and 700 µg/ml) were evaluated and the efficacy of the hyperthermia treatment on HeLa cells was obtained with 700 µg/ml of NPs (Figure S3). MTT assay on cells treated only with G-M48, but not exposed to MFH, showed viability of 95.56% while cells treated with 700 µg/mL NPs and exposed to MFH showed a viability of 72.57%.

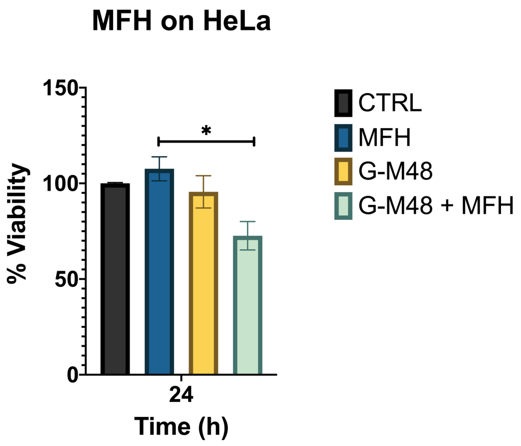

Figure S3. MTT assay in HeLa cells after MFH treatment.

Intratumoral localization of G-M48

In order to observe the distribution of NPs inside the tumor, T_2 weighted MR images were acquired before and after intratumoral injection of 50 µl G-M48 (1.2 mg Fe/ml) in mice. Representative images are reported in Figure S4 (a-b). Areas of signal drop were detected within the tumor mass (arrow in Figure S4 (b)) and in the subcutaneous region (asterisks in Figure S4 (b)). After MRI, the presence of G-M48 in the tumor tissues was confirmed by histological analysis. Prussian Blue staining of tumor tissue showed the presence of iron: several blue spots were detected inside the mass (Figure S4 (c)) and at the periphery of the tumour (Figure S4 (d)), corresponding to the dark areas detected in MRI.

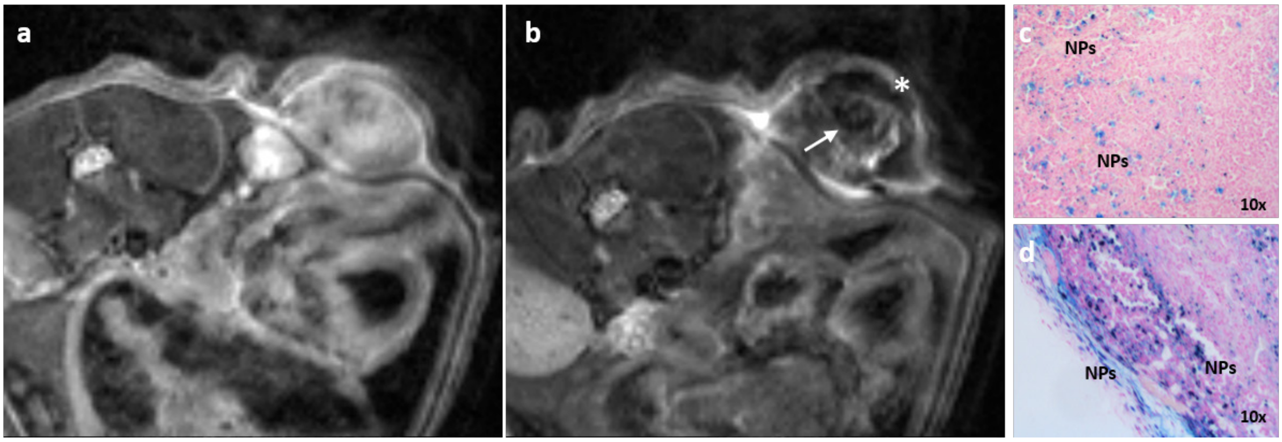

Figure S4. T_2 weighted MR images of tumor mass before (a) and after (b) intratumoral injection of G-M48 (1.2mg Fe/mL) in mice. The distribution of G-M48 is clearly visible (b) as hypointense areas (arrow and asterisks).The tumour mass was excised for histological examination with Prussian Blue staining; representative histological slices from the excised tumour tissue are reported in frames c-d: blue stained areas inside the tumour tissue (c) and at the tumour periphery (d) confirmed the presence of iron (magnification ×10).

Histological examination of tumor mass treated with G-M48

Six days after G-M48 intratumoral injection the tumor volume of mice treated with G-M48 increases of 179.51% ± 46.23 respect to the day of NPs injection. Representative MR images of mice treated with G-M48 at Day 0 and Day 6 is reported in Figure S6a. Histological analysis reveals the presence of NPs (b) and the preservation of tumor tissue (c). At day 6, the tumour masses were excised for histological examination. Figure S5 shows the Prussian Blue (b) and H&E (c) staining of the tumour tissue. Prussian Blue staining reveals several blue spots inside the tumor, proving the presence of iron inside the mass (Figure S5 (b)). Finally, mice treated with G-M48

don't exhibit evident modifications in the tumour tissue with a good preservation of cellular morphology, as shown in Figure S5 (c).

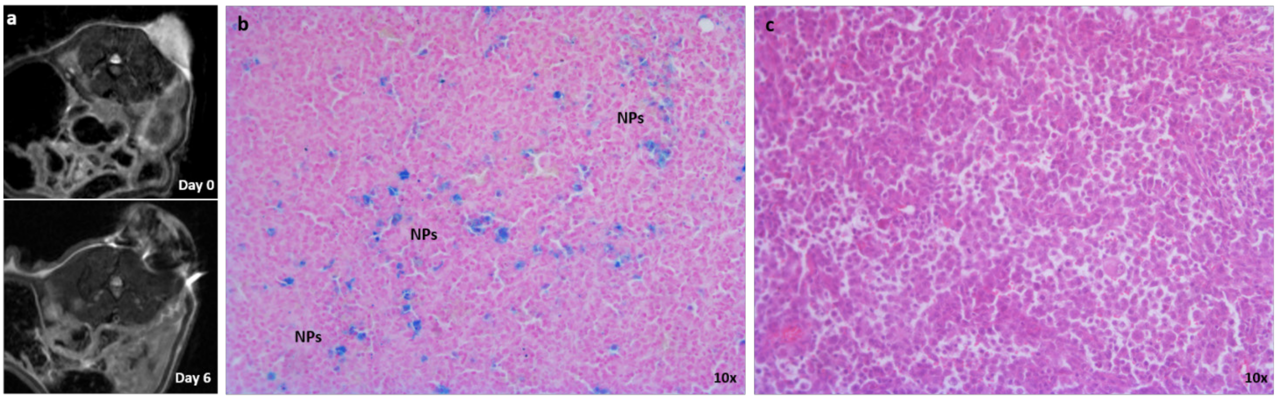

Figure S5. a) Representative MR images of mice treated with G-M48 at Day 0 and Day 6. The tumor growth is comparable to the saline group. (b-c) Histological analysis of tumor mass at day 6. Prussian Blue staining reveals the presence of iron (b), while H&E staining shows the preservation of tumor tissue (c).

References

[1] Cornell RM, Schwertmann U, The Iron Oxides: Structure, Properties, Reactions, Occurrences and Uses. Wiley-VCH. 2003.
